# Reliability and Accuracy of 2D Photogrammetry: A Comparison With Direct Measurement

**DOI:** 10.3389/fpubh.2021.813058

**Published:** 2022-01-25

**Authors:** Yin Cheng Lim, Ameerah Su'ad Abdul Shakor, Rafiza Shaharudin

**Affiliations:** ^1^Environmental Health Research Centre, Institute for Medical Research, National Institutes of Health, Ministry of Health of Malaysia, Shah Alam, Malaysia; ^2^Faculty of Medicine, Department of Social and Preventive Medicine, University of Malaya, Kuala Lumpur, Malaysia

**Keywords:** 2D photogrammetry, direct measurement, accuracy, facial anthropometric measurements, reliability

## Abstract

**Objective:**

Facial anthropometric data is important for the design of respirators. Two-dimensional (2D) photogrammetry has replaced direct anthropometric method, but the reliability and accuracy of 2D photogrammetry has not been quantified. This study aimed to assess inter-rater reliability of 2D photogrammetry and to examine the reliability and accuracy of 2D photogrammetry with direct measurement.

**Design:**

A cross-sectional study.

**Setting:**

Malaysia.

**Participants:**

A subset of 96 participants aged 18 and above.

**Primary and secondary outcomes:**

Ten facial dimensions were measured using direct measurement and 2D photogrammetry. An assessment of inter-rater reliability was performed using intra-class correlation (ICC) of the 2D images. In addition, ICC and Bland-Altman analyses were used to assess the reliability and agreement of 2D photogrammetry with direct measurement.

**Results:**

Except for head breadth and bigonial breadth, which were also found to have low inter-rater reliability, there was no significant difference in the inter-rater mean value of the 2D photogrammetry. The mean measurements derived from direct measurement and 2D photogrammetry were mostly similar. However, statistical differences were noted for two facial dimensions, i.e., bizygomatic breadth and bigonial breadth, and clinically the magnitude of difference was also significant. There were no statistical differences in respect to the remaining eight facial dimensions, where the smallest mean difference was 0.3 mm and biggest mean difference was 1.0 mm. The ICC showed head breadth had poor reliability, whilst Bland-Altman analyses showed seven out of 10 facial dimensions using 2D photogrammetry were accurate, as compared to direct measurement.

**Conclusion:**

Only certain facial measurements can be reliably and accurately measured using 2D photogrammetry, thus it is important to conduct a reliability and validation study before the use of any measurement methods in anthropometric studies. The results of this study also suggest that 2D photogrammetry can be used to supplement direct measurement for certain facial dimensions.

## Background

Craniometry is a specific component of anthropometry that focuses on the measurement of the anatomical size of the head and face of living subjects. It is widely applied in orthodontic and reconstructive surgery, forensics, and the design of helmets, masks, eyeglasses and respirators (1). Numerous local anthropometric studies have been undertaken to achieve various objectives for the different needs of a range of target groups such as preschool children (2), young adults (3) and older persons (4), and such studies include some that have focused on the facial anthropometry of the Malaysian population (5–10). Many of these studies highlight the importance of incorporating ergonomic principles into design to ensure end-products fit with the body conditions and sizes of the target users.

Several methods can be used to measure facial soft tissues. These include manual anthropometry (11, 12) and two-dimensional (2D) (5, 10, 13–16) and 3-dimensional (3D) (8, 17, 18) imaging techniques. Manual anthropometry takes direct measurements from the subject using sliding and spreading callipers, flexible measuring tapes and protractors. The main advantages of this method are that it is non-invasive and low cost. However, despite being considered the gold standard for facial measurement, it has some disadvantages; for example, it is time consuming and it depends on the participant's compliance for reliable results. Furthermore, it is investigator dependent, meaning that there is a possibility that the investigator may apply too much pressure on the equipment during measurement, which may distort soft tissue and introduce measurement errors.

Nowadays, 2D and 3D measurement techniques are commonly used to measure human anthropometric characteristics. The 2D imaging technique provides a snapshot of an object, thus it requires the participant's cooperation during image acquisition. Despite evidence to show that the 3D imaging technique is more accurate (19, 20), the 2D option is still preferred because it is cheap, non-invasive, less time consuming and can be conducted on the ground, as in population surveys.

A number of studies have compared the performance of different anthropometry methods (19, 21–25). For example, one study that investigated the difference in human skull measurements by comparing conventional cephalometric radiographs against 3D measurements on 3D models found that measurements of the same skull can differ significantly (21). Likewise, another study also noted significant differences in facial dimensions when using 2D and 3D imaging techniques, and concluded that the two facial anthropometry methods cannot be equivalently used (22). Conversely, other studies that compared 3D with 2D (19, 23, 24) and 3D with direct measurement (19, 25, 26) found that the methods produced comparable results in terms of identifying facial soft-tissue landmarks.

Although 2D images have been used widely for facial tissue analysis (5, 10), evidence demonstrating the accuracy of 2D photogrammetry in measuring facial dimensions is lacking and that which does exist shows contradicting results. While some studies have shown that different anthropometry methods can be used interchangeably (19, 24, 27), other studies have revealed otherwise (21, 22). Nevertheless, studies that have examined the reliability and agreement of 2D photogrammetry in measuring facial dimensions, as compared to the gold standard manual method are still limited (19, 20, 28). Thus, this study aimed to determine the inter-rater reliability of 2D photogrammetry in measuring facial anthropometry, as well as the reliability and agreement of 2D photogrammetry, as compared to direct measurement. The significance of this validation study is that it will be used for a future population nationwide study to help us to develop own bivariate and Principal Component Analysis (PCA) panels, which is critical for the development of respirator.

## Methodology

### Study Design

A cross-sectional study was conducted among a subset of participants aged 18 and above who were involved in the National Health and Morbidity Survey (NHMS) Malaysia 2020. NHMS 2020 was a national population-based survey aimed at determining seroprevalence of COVID-19, hepatitis B and C in Malaysia. Representative samples were selected randomly from 2000 living quarters in selected Enumeration Blocks (EBs) using a two stage stratified sampling from the Department of Statistics, Malaysia (29). All household members who consented and fit the inclusion criteria were recruited into the survey. For this validation study, a subset of 96 respondents was conveniently selected from one district (Banting, Selangor) in West Malaysia and one district (Tawau, Sabah) from East Malaysia. Participants with a history of previous facial surgery, dental or facial deformity, and those with a beard or moustache were excluded from participating.

### Data Collection

The 10 facial dimensions listed in Table 1 were measured because they are critical for the development of respirators (30). A measurer's manual was created prior to the field investigation. The measurer was trained until the measurement errors were less than what was allowed. Usually, the allowable error margin was set at 2 mm for all the dimensions measured (28, 31). Prior to image acquisition, direct measurements were taken.

**Table 1:** Description, definition, and diagram of measurements (30).

**Dimension**	**Description**	**Diagram**
1. Bigonial breadth	Distance between the right and left gonion
		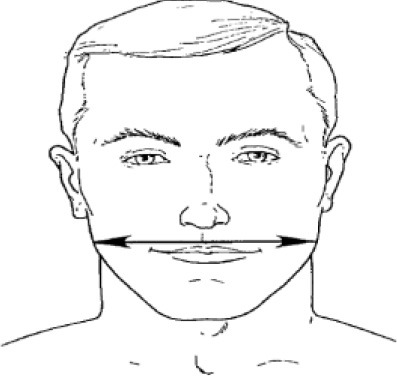
2. Bizygomatic breadth	Maximum horizontal breadth of the face
		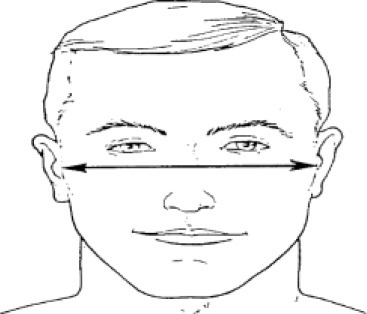
3. Head breadth	Maximum horizontal breadth of the head
		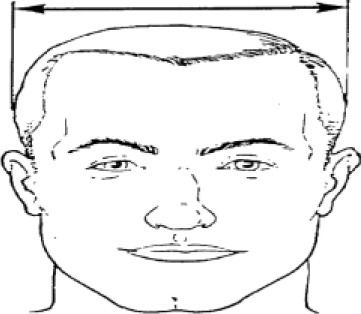
4. Interpupillary distance	Distance between the centre of pupil
		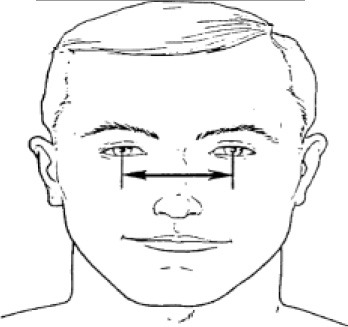
5. Menton-sellion length	Distance between the menton and the sellion
		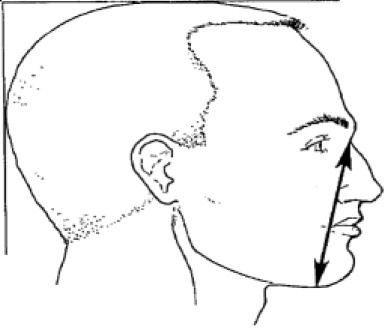
6. Minimum frontal breadth	Distance between the right and left frontotemporal
		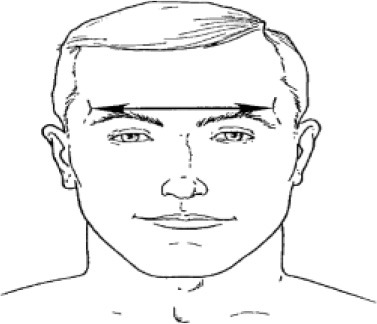
7. Nasal root breadth	Horizontal breadth of nose at the sellion
		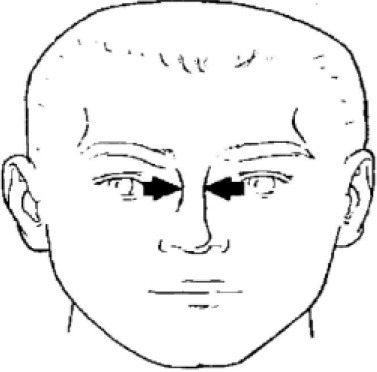
8. Nose breadth	Distance between the right and left alare
		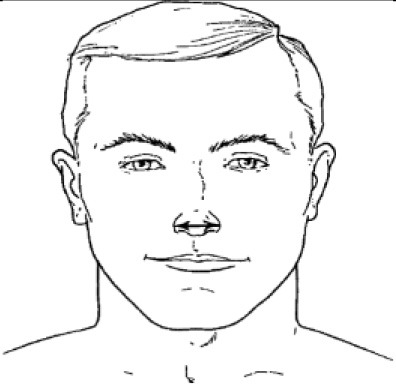
9. Nose protrusion	Distance between the pronasale and the subnasale
		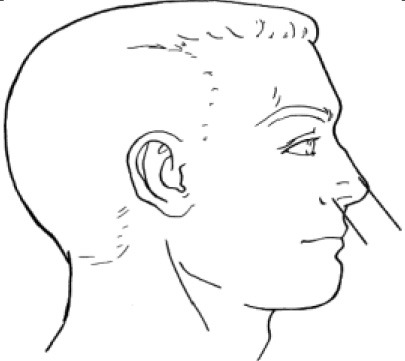
10. Subnasale-sellion length	Distance between the subnasale and the sellion
		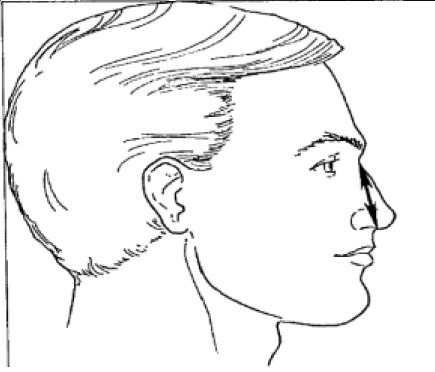

### Direct Measurement Procedure

The 10 selected morphological points were located by inspection and/or palpation in accordance with the 1988 Anthropometric Survey of the US Army Personnel Project (31). Spreading callipers were used to measure head breadth, zygomatic breadth and bigonial breadth, whereas sliding callipers were used for the remaining seven facial dimensions. During the measurement process, the investigators endeavoured to ensure that the participants were relaxed and seated with a natural head position and relaxed lips.

### Photographic Set-Up

The participants' images were captured using a 20.0-megapixel digital camera (Canon IXUS 190, Tokyo, Japan) positioned on a tripod (Manfrotto MKCOMPACTLT-BK, Cassola, Italy) at a fixed distance of 1.0 metre. The tripod maintained the stability and correct height of the camera according to each participant's height. Participants were requested to wear a surgical cap to remove hair strands from their face and ears when needed. The frontal bony landmarks on their face were labelled with stickers. Before image capture, the participants were asked to look straight ahead holding their head in a neutral position without flexing or extending the neck, and to not smile or frown. Also, the head was kept in a posture so that the optical axis of the camera lens would pass through the Frankfurt plane of the head (32).

For each participant, one anterior and one lateral photo were taken. A blue screen was used as the background in order to create sufficient contrast with the colour of the skin. The camera height was adjusted based on the height of the subject's ear from the floor. For calibration purposes, a metric-scale ruler was placed above the forehead of the subject for the image taken from the anterior view (Supplementary e-Figure 1). On the other hand, the ruler was placed perpendicular to the nose of the subject for the image taken from the lateral view (Supplementary e-Figure 2). Each image was checked immediately after it was obtained to ensure absence of acquisition errors such as imaging artefacts, blurring, absence of surface data, poor orientation, closed eyes, and lack of neutral facial expression. Images with the incorrect characteristics were discarded, and new images were obtained to ensure that they met the established requirements.

All images were captured in JPEG format and were transferred to a computer after each day of shooting. The anthropometric dimensions were calculated using the software package Digimizer version 5.4.4. This software is very useful for analysing images as it is very flexible and simple to use. Its capabilities include providing the user with the ability to set contrast and brightness, change background images, change images to grayscale mode, measure angles, determine the centre of the segment and reduce image noise (33).

### Inter-Rater Reliability of 2D Photogrammetry

For the inter-rater reliability assessment, the measurements made by the first observer were compared with those carried out by the second observer on the same photo images at a minimum of a 3-week interval, with no landmarks saved after the first measurement.

### Statistical Analysis

The analyses were conducted using SPSS version 26 and MedCalc version 19.8. Normal distribution of the data was evaluated using the Shapiro-Wilk test. The results showed that none of the variables violated the normality distribution. To evaluate the inter-rater measurement reproducibility of 2D images, a different observer took measurements using the same method as the first observer, and results were evaluated using paired sample *t*-test and intra-class correlation (ICC) coefficient. Next, paired sample *t*-test and ICC were also conducted to determine the mean differences and reliability between direct measurement and the average of 2D photogrammetry methods.

ICC provides information on the ability to differentiate variations between participants and measurement. The ICC was defined as the ratio of variance among participants (participant variability) over the total variance (participant variability, observer variability and measurement variability). The ICC value ranges between 0 (no reliability) and one (perfect reliability). In line with prior research, in this study < 0.4 indicates poor reliability, 0.4–0.75 indicates moderate reliability, and ≥ 0.75 indicates excellent reliability (34).

The degree of agreement between the two methods was further evaluated using Bland-Altman analysis, where the difference between the measurements was plotted against the average of the two measurements. The plot generates three horizontal reference lines that are superimposed on a scatterplot: one line represents the average difference between the measurements, and the upper and lower lines mark the two-standard deviation (±2 SD) from the mean differences. In a Bland-Altman analysis, two criteria need to be met to establish that the two measurement methods are comparable. First, the mean differences should be small and close to 0. Second, the SD of this difference should be small (35). However, there are no guidelines on how narrow the limit of agreement needs to be before the two methods can be considered interchangeable.

For all the statistical analyses, the methods were considered to be in good agreement and interchangeable at an arbitrary value of 2 mm between two observers and two methods (28). The statistical significance level was set as *p* < 0.005 for all statistical analysis.

### Patient and Public Involvement

The study participants were not involved in the development of this study. The results of the study were not shared with the participants.

## Results

A total of 96 participants participated in this study, of whom 51 (53.1%) were female. The mean age of the participants was 43.3 ± 16.9 years old and they were predominantly of Malay ethnicity (60, 62.5%).

A reproducibility assessment was conducted to determine the mean differences and the level of reliability of 2D photogrammetry between two observers. The mean values between the two observers for the abovementioned 10 facial dimensions revealed no significant difference, except for bigonial breadth (8.1 mm) and head breadth (11.2 mm) (Table 2). The inter-rater ICC scores for the eight facial dimensions of 2D photogrammetry varied from 0.66 (95% CI 0.50–0.76) for bizygomatic breadth to 0.99 (95 % CI 0.98–0.99) for minimum frontal breadth, except for bigonial breadth (ICC: 0.16, 95 % CI: −0.25 to 0.44) and head breadth (ICC: 0.03, 95% CI: −0.46 to 0.35).

**Table 2:** Summary of anthropometric statistics between observers of the 2D photogrammetry and inter-rater reliability coefficient of 2D photogrammetry.

**Dimensions**	**Observer A Mean ±SD**	**Observer B Mean ±SD**	**Mean differences ±SD**	**95% CI of mean differences**	***P*** **-value**	**ICC**	**95% CI**
1. Bizygomatic breadth*	140.2, 11.9	141.0, 9.8	0.8, 12.4	−3.1 to 1.5	0.468	0.66	0.50 to 0.76
2. Minimum frontal breadth	98.9, 9.1	99.0, 9.1	0.1, 1.5	−0.4 to 0.2	0.719	0.99	0.99 to 0.99
3. Bigonial breadth†	118.9, 13.0	126.9, 11.58	8.1, 12.4	−10.6 to −5.6	<0.005	0.16	−0.25 to 0.44
4. Menton-sellion length	115.8, 9.2	115.9, 9.3	0.1, 2.1	−0.5 to −0.4	0.776	0.99	0.98 to 0.99
5. Interpupillary distance	64.4, 3.8	64.6, 3.9	0.1, 1.1	−0.4 to −0.1	0.248	0.98	0.97 to 0.99
6. Head breadth†	149.3, 18.3	160.6, 9.7	11.2, 19.5	−15.2 to −0.7	<0.005	0.03	−0.46 to 0.35
7. Nose protrusion	17.1, 2.3	17.1, 2.2	0.1, 1.1	−0.2 to 0.2	0.865	0.94	0.91 to 0.96
8. Nose breadth	42.2, 3.5	42.3, 3.6	0.1, 1.1	−0.3 to 0.1	0.343	0.98	0.97 to 0.99
9. Nasal root breadth	18.9, 2.7	18.5, 2.3	0.4, 1.8	−0.1 to 0.7	0.060	0.85	0.78 to 0.91
10. Subnasal-sellion length	47.3, 4.6	47.5, 4.6	0.2, 2.0	−0.6 to 0.2	0.411	0.95	0.93 to 0.98

* *Moderate reliability with wide CI*.

† *Low reliability with wide CI*.

*CI, confidence interval; ICC, intraclass correlation; SD, standard deviation*.

The mean differences between the direct and the 2D photogrammetry measurements were within 2.0 mm, except for bizygomatic breadth and bigonial breadth (Table 3). The largest mean differences were observed in bigonial breadth (9.3 mm), followed by bizygomatic breadth (3.3 mm). The smallest mean difference between the two methods was found in nose protrusion (0.4 mm) and nose breadth (0.4 mm).

**Table 3:** Summary of anthropometric statistics between direct measurement and 2D photogrammetry.

**Dimensions**	**Direct measurement Mean ±SD**	**2D photogrammetry Mean ±SD**	**Mean differences ±SD**	**95% CI of mean differences**	***P*** **-value**
1. Bizygomatic breadth	137.3, 9.6	140.6, 9.3	3.3, 5.5	2.2 to 4.4	<0.005
2. Minimum frontal breadth	98.4, 9.8	99.0, 9.0	0.6, 2.9	0.02 to 1.2	0.040
3. Bigonial breadth	113.5, 10.4	122.8, 11.1	9.3, 5.3	8.3 to 10.4	<0.005
4. Menton-sellion length	116.0, 10.8	115.8, 9.2	1.0, 6.4	−2.3 to 0.3	0.124
5. Interpupillary distance	64.5, 3.8	63.9, 4.2	0.7, 2.4	0.1 to 1.1	0.012
6. Head breadth	154.6, 7.8	154.9, 10.9	0.3, 9.9	1.7 to 2.4	0.733
7. Nose protrusion	16.7, 2.6	17.1, 2.2	0.4, 2.1	0.1 to 0.9	0.049
8. Nose breadth	42.6, 3.8	42.2, 3.5	0.4, 2.3	−0.1 to 0.9	0.078
9. Nasal root breadth	18.2, 3.6	18.7, 2.4	0.5, 2.3	0.1 to 1.0	0.028
10. Subnasal-sellion length	47.7, 4.9	47.4, 4.5	0.3, 1.3	−0.5 to −0.1	0.044

*CI, confidence interval; SD, standard deviation*.

The reliability of using 2D photogrammetry and direct measurement for all measured dimensions varied from ICC = 0.81 (nose protrusion) to 0.99 (subnasal sellion length), except for head breadth [ICC: 0.36, 95% confidence interval (CI): 0.05–0.58] (Table 4). The highest ICC score was noted for subnasal sellion length (ICC: 0.99, 95% CI: 0.98–0.99), followed by menton sellion length (ICC: 0.98, 95% CI: 0.97–0.99) and minimum frontal breadth (ICC: 0.98, 95% CI: 0.97–0.99).

**Table 4:** Reliability coefficient between direct measurement and 2D photogrammetry.

**Dimensions**	**ICC**	**95% CI**
1. Bizygomatic breadth	0.84	0.76–0.90
2. Minimum frontal breadth	0.98	0.97–0.99
3. Bigonial breadth	0.91	0.86–0.94
4. Menton-sellion length	0.98	0.97–0.99
5. Interpupillary distance	0.90	0.92–0.97
6. Head breadth*	0.36	0.05–0.58
7. Nose protrusion	0.81	0.72–0.88
8. Nose breadth	0.92	0.88–0.95
9. Nasal root breadth	0.83	0.75–0.90
10. Subnasal-sellion length	0.99	0.98–0.99

* *Low reliability with wide CI*.

*CI, confidence interval; ICC, intraclass correlation*.

Supplementary e-Figure 3a to Supplementary e-Figure 3j show the level of agreements between direct measurement and 2D photogrammetry for 10 facial dimensions according to Bland-Altman plots (Supplementary e-Figure 3). The Y axis displayed the mean difference between two methods, whereas the X axis showed the mean of two different method. Ninety-five percentage CI of limit of agreement was chosen to demonstrate the error bars for both the upper and lower limit of agreement. Seven facial dimensions showed a high degree of agreement between the two methods, i.e., minimum frontal breadth, subnasal sellion length, menton sellion length, interpupillary distance, nose protrusion, nose breadth and nasal root breadth. Poor agreement with a wide 95% CI was found for bigonial breadth (mean = 9.4, 95% CI: −0.9 to 19.6), bizygomatic breadth (mean = 3.3, 95% CI: −7.5 to 14.2) and head breadth (mean = 0.3, 95% CI: −19.0 to 19.7).

## Discussion

This study evaluated the reliability and accuracy of 2D photogrammetry, as compared to direct measurement which has been accepted as the gold standard. Our study showed that three facial dimensions, i.e., bigonial breadth, bizygomatic breadth, and head breadth, cannot be measured reliably and accurately using the 2D photogrammetry method. This was because, there were poor inter-rater reliability of 2D photogrammetry as well as between two different measurement methods for bigonial breadth and head breadth. There was also significant difference in the mean values between the two methods for bizygomatic breadth and bigonial breadth. Thus, only seven out of 10 facial dimensions can be measured reliably and accurately using 2D photogrammetry. The main reason for inaccurate head breadth may be the demography of respondents in this study. In Malaysia, the predominant religion is Islam and most female Muslims wear the hijab as a demonstration of their faith following the requirements of their religion. However, even without the hijab, the head breadth cannot be measured accurately because of varying hair thickness. Because of the limitations of 2D photogrammetry, it is also quite impossible to view zygomatic and gonial landmarks from the anterior view in 2D photogrammetry, even after marking the bony landmarks with stickers. The remaining seven dimensions showed no difference in terms of mean value and had a high level of agreement according to the ICC analysis.

The 2D photogrammetric method has been used widely by international (13–16) and local studies (5, 10). However, studies that compare 2D photogrammetry with direct measurement are scarce and have some limitations (19, 20, 28). There is also a lack of consensus among the existing studies. One study showed that 2D photogrammetry is not as accurate as the direct and 3D measurement methods for certain facial dimensions (19), while the two other studies showed that 2D photogrammetry is comparable to direct measurement (20, 28). Moreover, previous studies have mainly focused on oral maxillofacial dimensions, in contrast to our study, and none of the studies assessed the reliability and agreement of 2D photogrammetry with direct measurement simultaneously (20, 28). Furthermore, appropriate data analysis should be employed to confirm that tested and validated tools are both reliable and accurate.

Hence, the validation of the 10 facial dimensions considered in our study will be an important step in our future research, which aims to produce an anthropometric database of Malaysian head and facial measurements. The same 10 facial dimensions were used by the United States National Institute for Occupational Safety and Health in 2003 on 3997 civilian workers and by the Chinese government in 2008 on 3000 Chinese civilian workers to develop respirator fit test panels (30, 36). Respirator fit test panels provide an objective measurement for selecting representative human test samples based on their facial dimensions for use in research, testing, certification and most importantly for respirator development.

Likewise, the 10 critical facial dimensions measured in our study can be used to develop two respirator fit test panels, i.e., a bivariate panel using face length and face width and a PCA panel using all 10 facial dimensions. The bivariate panel is simpler to use than the PCA panel. However, the inclusion of the eight additional facial measurements allows the PCA panel to apply better criteria to exclude the use of extreme face sizes. These 10 dimensions have been found to be associated with respirator fit and leakage and can predict the remaining face dimensions well (1). Moreover, the study in the United States showed that respirators designed to fit PCA panel are expected to accommodate more than 95% of current US civilian workers (30).

We acknowledge that our study has some limitations. First, direct measurements were only measured by one observer, thus reliability of this method cannot be calculated, as compared to the 2D photogrammetry measurements. However, we believe that the measurement errors in the direct measurement procedure were minimal in view of training that was conducted prior to the validation part of the study. Even though the facial dimensions were not measured using the 3D photogrammetry method, which has been found to be more accurate, direct measurement or 2D photogrammetry are more feasible for a nationwide population survey, especially in low- and middle-income countries. However, the disadvantages of the 2D technique include measurement errors due to subjective analysis, magnification errors, parallax, variation in lighting, and variation in head orientation.

On the other hand, the novelty of our work lies in the robust validation analysis that we undertook to validate the results generated by 2D photogrammetry against the gold standard of direct measurement. In addition to comparing the mean values of these two methods, we also used ICC and Bland-Altman Limit of Agreement analysis. The Bland-Altman Limit of Agreement and the ICC are the most popular methods to investigate statistical agreement and to assess the reliability of medical instruments, respectively (37). Agreement and reliability parameters are equally important in determining the quality of the applied method and these two parameters have not been assessed together in previous validation studies (19, 20). It is important to note that a method with good reliability will not be useful if it is not in good agreement with and vice versa. The other strength of our study lies in the reporting of the CI value when using the limits of agreement approach, as this means that the data can be generalised to a larger population. Moreover, the advantage of using the Bland-Altman approach is that it can reveal both systematic errors (bias) and random errors (limit of agreement) (38).

## Conclusion

This study reveals that only seven out of 10 facial measurements can be measured reliably and accurately using 2D photogrammetry, thus it is important that a validation and reliability study is conducted before the use of any measurement methods in anthropometric studies. The results of this study also suggest that, given its practical benefits of being inexpensive, non-invasive, operator dependent and less time consuming, 2D photogrammetry can be used to supplement direct measurement for facial dimensions. Our future study, which will take place during the COVID-19 pandemic, will use a combination of direct measurement and 2D photogrammetry to create an anthropometric database of Malaysian head and facial measurements from over 3,000 participants. The use of 2D photogrammetry can also help to reduce exposure between observers and participants. The findings also indicate the important role that 2D photogrammetry can play in assessing certain facial morphologies in countries that have limited 3D scanner resources. Lastly, future studies to compare and validate the output of 2D photogrammetry against direct measurement in respect of other facial dimensions are also warranted to ensure that more of the dimensions can be measured in this way and it will be both accurate and reliable.

## Data Availability Statement

The original contributions presented in the study are included in the article/Supplementary Materials, further inquiries can be directed to the corresponding author.

## Ethics Statement

The studies involving human participants were reviewed and approved by Medical Research and Ethics Committee (NMRR-20-1217-55489). The patients/participants provided their written informed consent to participate in this study. Written informed consent was obtained from the individual(s) for the publication of any potentially identifiable images or data included in this article.

## Author Contributions

The study conception was by YL and RS. YL and RS designed the study. YL collected the data. YL, AA, and RS conducted the statistical analysis and interpreted the results. YL and AA drafted the manuscript. All authors have read and approved the final version of the submitted manuscript.

## Funding

This funding for this study (NMRR-20-1217-55489 [20-044]) and the funding for publication were from the National Institutes of Health, Ministry of Health, Malaysia.

## Conflict of Interest

The authors declare that the research was conducted in the absence of any commercial or financial relationships that could be construed as a potential conflict of interest.

## Publisher's Note

All claims expressed in this article are solely those of the authors and do not necessarily represent those of their affiliated organizations, or those of the publisher, the editors and the reviewers. Any product that may be evaluated in this article, or claim that may be made by its manufacturer, is not guaranteed or endorsed by the publisher.

## Abbreviations

95%CI: 95% Confidence Interval
2D: 2-Dimensional
3D: 3-Dimensional
ICC: Intraclass correlation
NHMS: National Health and Morbidity Survey
NIOSH: National Institute for Occupational Safety and Health
PCA: Principal Component Analysis
SD: Standard deviation
SE: Standard error.

## References

[1] Zhuang Z Coffey CC Ann RB. The effect of subject characteristics and respirator features on respirator fit. J Occup Environ Hyg. (2005) 2:641–9. 10.1080/15459620500391668 16298949

[2] Bari SB Othman M Salleh NM. Foot anthropometry for shoe design among preschool children in Malaysia. Pertanika J Soc Sci Humanit. (2010) 18:69–79.

[3] Karmegam K Sapuan S Ismail M Ismail N Bahri MS Shuib S. Anthropometric study among adults of different ethnicity in Malaysia. Int J Phys Sci. (2011) 6:777–88. 10.5897/IJPS10.31027411716

[4] Rashid S Hussain M Yusuff R. Designing homes for the elderly based on the anthropometry of older Malaysians. Asian J Gerontol Geriatr. (2008) 3:75–83.

[5] Lin CS Shaari R Alam MK Rahman SA. Photogrammetric analysis of nasolabial angle and mentolabial angle norm in Malaysian adults. Bangladesh J Medical Sci. (2013) 12:209–19. 10.3329/bjms.v12i2.14951

[6] Ngeow WC Aljunid S. Craniofacial anthropometric norms of Malays. Singapore Med J. (2009) 50:525. 19495526

[7] Ngeow WC Aljunid S. Craniofacial anthropometric norms of Malaysian Indians. Indian J Dent Res. (2009) 20:313. 10.4103/0970-9290.57372 19884715

[8] Othman SA Majawit LP Wan Hassan WN Wey MC Mohd Razi R. Anthropometric study of three-dimensional facial morphology in Malay adults. PLoS ONE. (2016) 11:e0164180. 10.1371/journal.pone.0164180 27706220 PMC5051712

[9] Wai MM Thwin SS Yesmin T Ahmad A Adnan AS Hassan AA. Nasofacial anthropometric study among university students of three races in Malaysia. Adv Anat Pathol. (2015) 2015. 10.1155/2015/7807562015

[10] Packiriswamy V Kumar P Rao KG. Photogrammetric analysis of palpebral fissure dimensions and its position in malaysian South Indian ethnic adults by gender. N Am J Med Sci. (2012) 4:458–62. 10.4103/1947-2714.101984 23112966 PMC3482776

[11] Du L Zhuang Z Guan H Xing J Tang X Wang L. Head-and-face anthropometric survey of Chinese workers. Ann Occup Hyg. (2008) 52:773–82. 10.1093/annhyg/men056 18765398

[12] Zhuang Z Landsittel D Benson S Roberge R Shaffer R. Facial anthropometric differences among gender, ethnicity, and age groups. Ann Occup Hyg. (2010) 54:391–402. 10.1093/annhyg/meq007 20219836

[13] Milosević SA Varga ML Slaj M. Analysis of the soft tissue facial profile of Croatians using of linear measurements. J Craniofac Surg. (2008) 19:251–8. 10.1097/scs.0b013e31815c9446 18216697

[14] Malkoç S Demir A Uysal T Canbuldu N. Angular photogrammetric analysis of the soft tissue facial profile of Turkish adults. Eur J Orthod. (2009) 31:174–9. 10.1093/ejo/cjn082 19064675

[15] Fernández-Riveiro P Smyth-Chamosa E Suárez-Quintanilla D Suárez-Cunqueiro M. Angular photogrammetric analysis of the soft tissue facial profile. Eur J Orthod. (2003) 25:393–9. 10.1093/ejo/25.4.393 12938846

[16] Beugre JB Diomande M Assi AR Koueita MK Vaysse F. Angular photogrammetric analysis and evaluation of facial esthetics of young Ivorians with normal dental occlusion. Int Orthod. (2017) 15:25–39. 10.1016/j.ortho.2016.12.015 28073627

[17] Verhoeven TJ Coppen C Barkhuysen R Bronkhorst EM Merkx MA Bergé SJ. Three dimensional evaluation of facial asymmetry after mandibular reconstruction: validation of a new method using stereophotogrammetry. Int J Oral Maxillofac Surg. (2013) 42:19–25. 10.1016/j.ijom.2012.05.036 22939875

[18] Erten O Yilmaz BN. Three-dimensional imaging in orthodontics. Turk J Orthod. (2018) 31:86–94. 10.5152/TurkJOrthod.2018.17041 30206567 PMC6124883

[19] Ghoddousi H Edler R Haers P Wertheim D Greenhill D. Comparison of three methods of facial measurement. Int J Oral Maxillofac Surg. (2007) 36:250–8. 10.1016/j.ijom.2006.10.001 17113754

[20] Kook MS Jung S Park HJ Oh HK Ryu SY Cho JH. A comparison study of different facial soft tissue analysis methods. J Craniomaxillofac Surg. (2014) 42:648–56. 10.1016/j.jcms.2013.09.010 24954528

[21] Van Vlijmen O Maal T Bergé S Bronkhorst E Katsaros C Kuijpers-Jagtman A. comparison between 2D and 3D cephalometry on CBCT scans of human skulls. Int J Oral Maxillofac Surg. (2010) 39:156–60. 10.1016/j.ijom.2009.11.017 20044238

[22] Anas I Bamgbose B Nuhu S. A comparison between 2D and 3D methods of quantifying facial morphology. Heliyon. (2019) 5:e01880. 10.1016/j.heliyon.2019.e01880 31338446 PMC6579906

[23] Ayaz I. Shaheen E. Accuracy and reliability of 2-dimensional photography versus 3-dimensional soft tissue imaging. Imaging Sci Dent. (2020) 50:15–22. 10.5624/isd.2020.50.1.15 32206616 PMC7078411

[24] Zogheib T Jacobs R Bornstein M Agbaje J Anumendem D Klazen Y. Comparison of 3D scanning versus 2D photography for the identification of facial soft-tissue landmarks. Open Dent J. (2018) 12:61. 10.2174/1874210601812010061 29492171 PMC5814946

[25] Joe PS Ito Y Shih AM Oestenstad RK Lungu CT. Comparison of a novel surface laser scanning anthropometric technique to traditional methods for facial parameter measurements. J Occup Environ Hyg. (2012) 9:81–8. 10.1080/15459624.2011.640557 22214207

[26] Weinberg SM Naidoo S Govier DP Martin RA Kane AA Marazita ML. Anthropometric precision and accuracy of digital three-dimensional photogrammetry: comparing the Genex and 3dMD imaging systems with one another and with direct anthropometry. J Craniofac Surg. (2006) 17:477–83. 10.1097/00001665-200605000-00015 16770184

[27] Habibi E Soury S Zadeh AH. Precise evaluation of anthropometric 2D software processing of hand in comparison with direct method. J Med Signals Sens. (2013) 3:256. 10.4103/2228-7477.128338 24696802 PMC3967428

[28] Dindaroglu F Kutlu P Duran GS Görgülü S Aslan E. Accuracy and reliability of 3D stereophotogrammetry: a comparison to direct anthropometry and 2D photogrammetry. Angle Orthod. (2016) 86:487–94. 10.2319/041415-244.1 26267357 PMC8601748

[29] Institute for Public Health. NHMS 2020 Malaysia: Ministry of Health. Kuala Lumpur (2020). Available online at: https://iku.moh.gov.my/nhms-2020

[30] Zhuang Z Bradtmiller B Shaffer RE. New respirator fit test panels representing the current U. S civilian work force. J Occup Environ Hyg. (2007) 4:647–59. 10.1080/15459620701497538 17613722

[31] Clauser C Tebbetts I Bradtmiller B McConville J Gordon CC. Measurer's handbook: US Army anthropometric survey, 1987–1988. Anthropology Research Project Inc Yellow Springs Oh. (1988). 10.21236/ADA20272128271959

[32] Borman H Ozgür F. A simple instrument to define the frankfurt horizontal plane for soft-tissue measurements of the face. Plast Reconstr Surg. (1998) 102:580–1. 10.1097/00006534-199808000-00057 9703107

[33] MedCalc Software Ltd. Digimizer (2021). Available online at: https://www.digimizer.com/

[34] Koo TK Li MY. A Guideline of Selecting and Reporting Intraclass Correlation Coefficients for Reliability Research. J Chiropr Med. (2016) 15:155–63. 10.1016/j.jcm.2016.02.012 27330520 PMC4913118

[35] Bland JM Altman DG. Statistical methods for assessing agreement between two methods of clinical measurement. Lancet. (1986) 1:307–10. 10.1016/S0140-6736(86)90837-8 2868172

[36] Chen W Zhuang Z Benson S Du L Yu D Landsittel D. New respirator fit test panels representing the current Chinese civilian workers. Ann Occup Hyg. (2009) 53:297–305. 10.1093/annhyg/men089 19174486

[37] Zaki R Bulgiba A Ismail R Ismail NA. Statistical methods used to test for agreement of medical instruments measuring continuous variables in method comparison studies: a systematic review. PLoS ONE. (2012) 7:e37908. 10.1371/journal.pone.0037908 22662248 PMC3360667

[38] Rafdzah Z Bulgiba A Ismail N. Method comparison studies in medicine. J Transl Med. (2013) 16:16–22. 10.22452/jummec.vol16no1.4

