# Supplementary figures and images for: Reliability and Accuracy of 2D Photogrammetry: A Comparison With Direct Measurement
Source: Front Public Health. 2022 Jan 25;9:813058. doi: 10.3389/fpubh.2021.813058 (PMC8826070; doi:10.3389/fpubh.2021.813058)

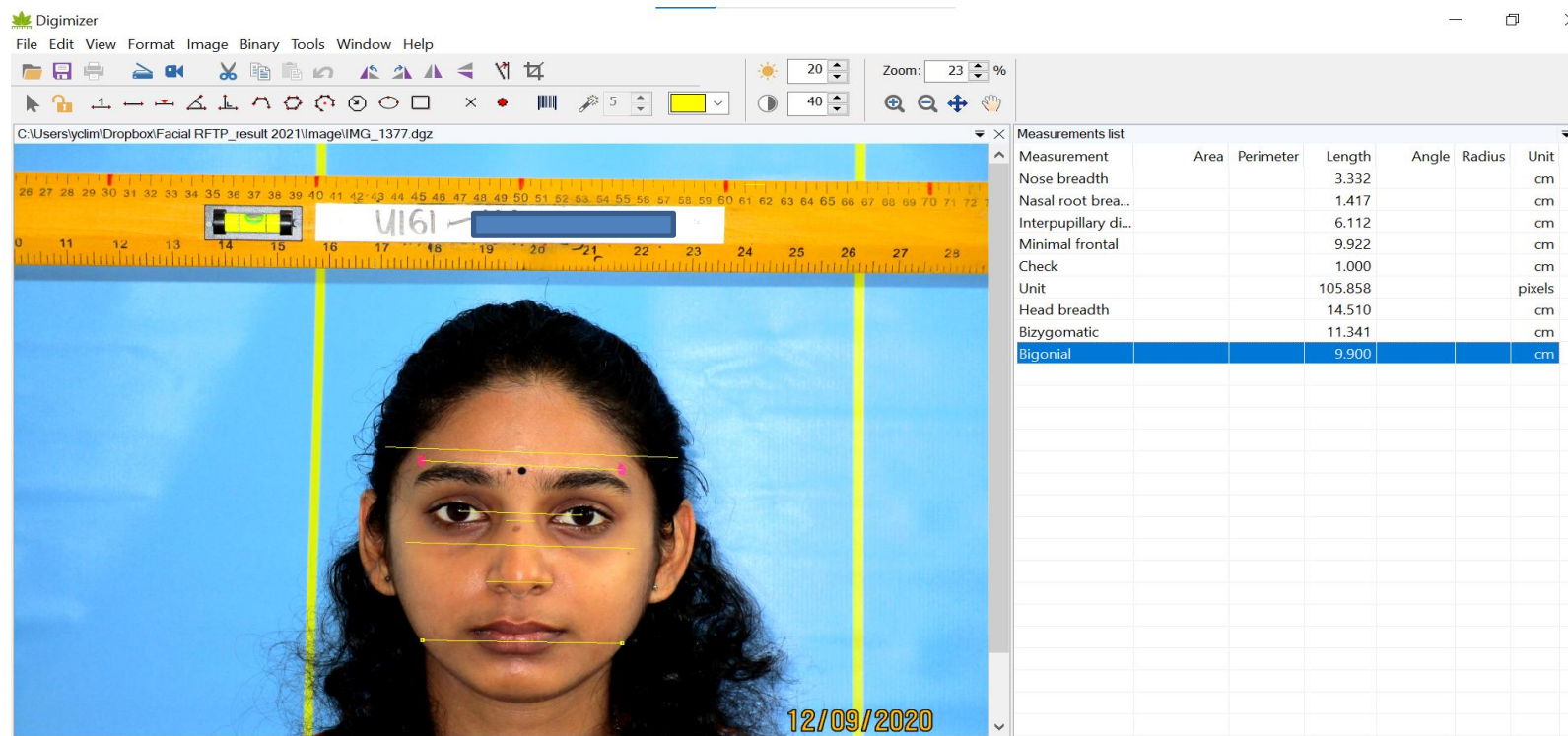

e-Figure 1 Measurement of the facial dimensions using Digimazer version 5.4.4 from anterior view

Supplement: Supplementary file 1 [file Data_Sheet_1.PDF]

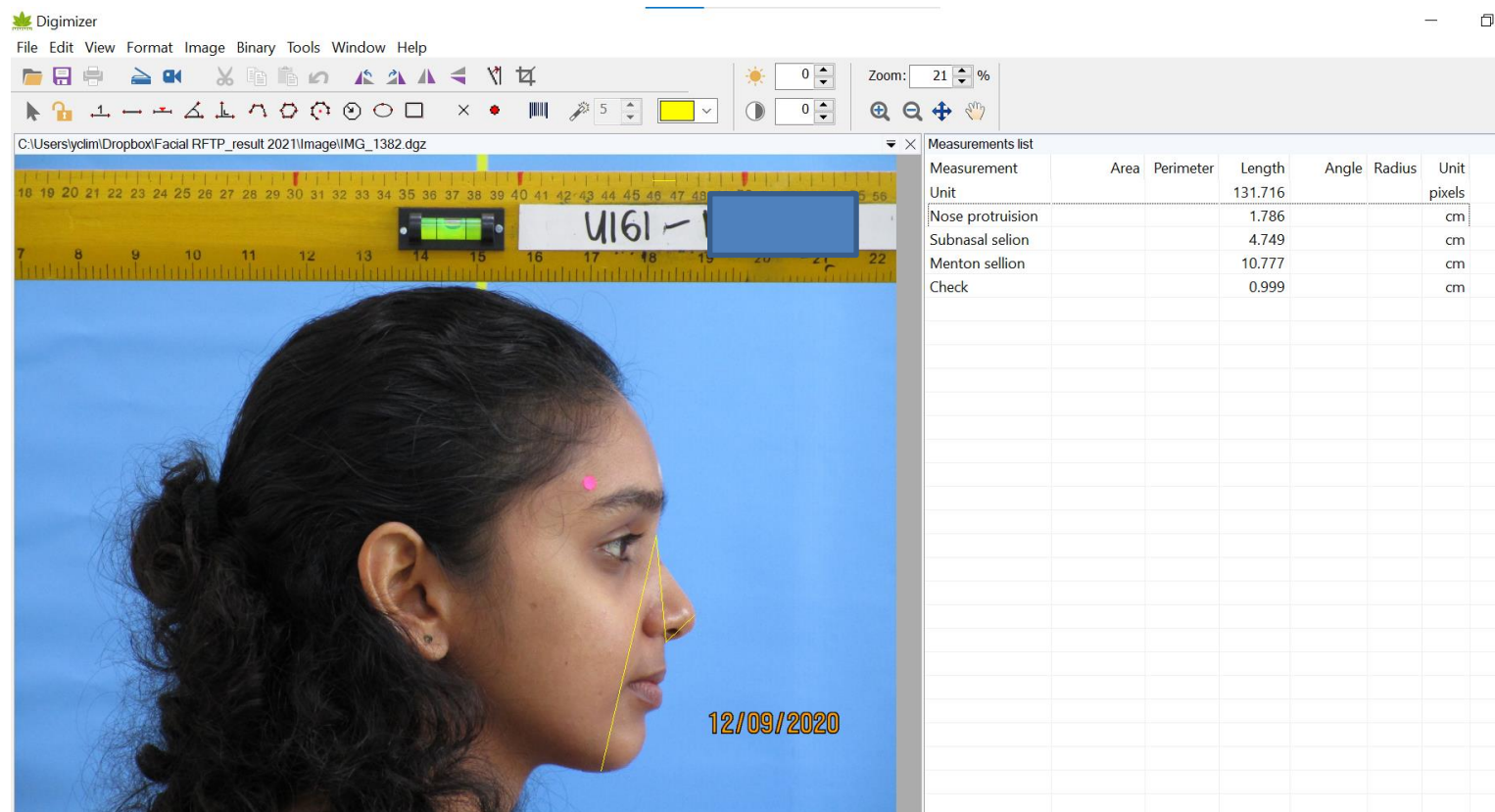

e-Figure 2 Measurement of the facial dimensions using Digimazer version 5.4.4 from lateral view

Supplement: Supplementary file 2 [file Data_Sheet_2.PDF]
