# Supplementary material for: Reliability and Accuracy of 2D Photogrammetry: A Comparison With Direct Measurement
Source: Front Public Health. 2022 Jan 25;9:813058. doi: 10.3389/fpubh.2021.813058 (PMC8826070; doi:10.3389/fpubh.2021.813058)
Supplement: Supplementary file 3 [file Data_Sheet_3.PDF]

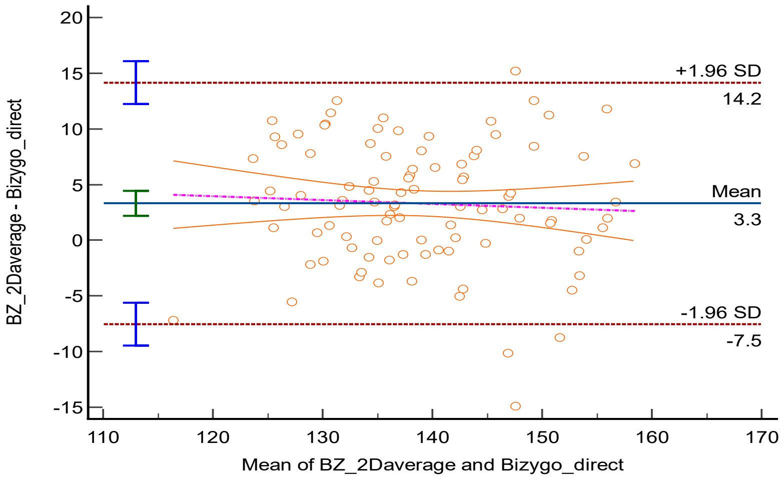

e-Figure 3a: Bland-Alman plot showed agreement between direct measurement and 2D photogrammetry for Bizygomatic breadth, and 95% limit of its agreement

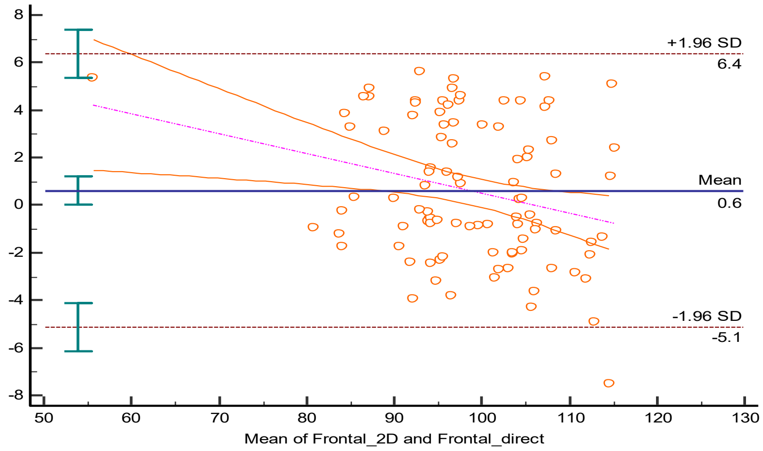

e-Figure 3b: Bland-Alman plot showed agreement between direct measurement and 2D photogrammetry for Minimal Frontal Breadth, and 95% limit of its agreement

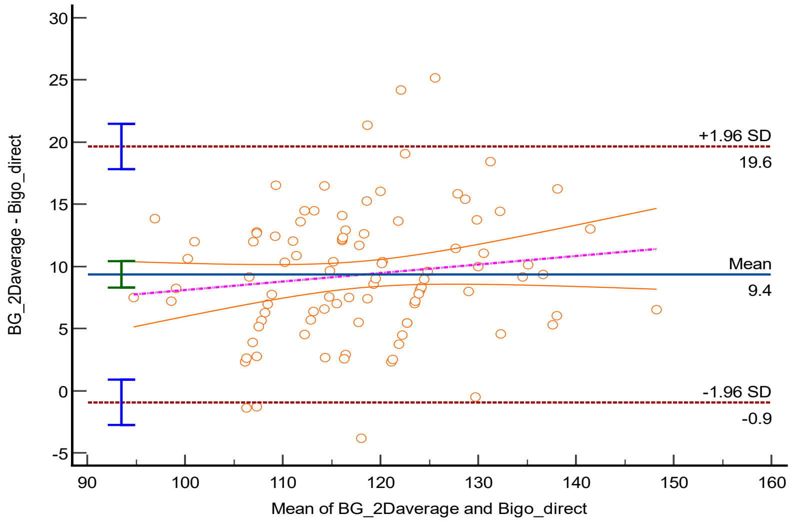

e-Figure 3c: Bland-Alman plot showed agreement between direct measurement and 2D photogrammetry for Bigonial Breadth, and 95% limit of its agreement

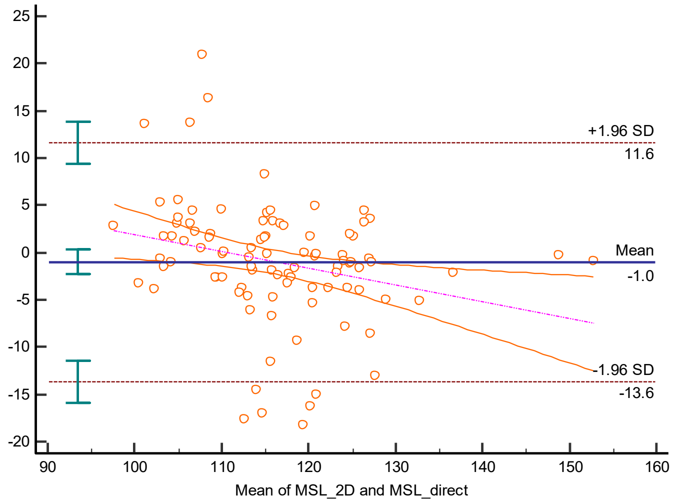

e-Figure 3d: Bland-Alman plot showed agreement between direct measurement and 2D photogrammetry for Menton-sellion Length, and 95% limit of its agreement

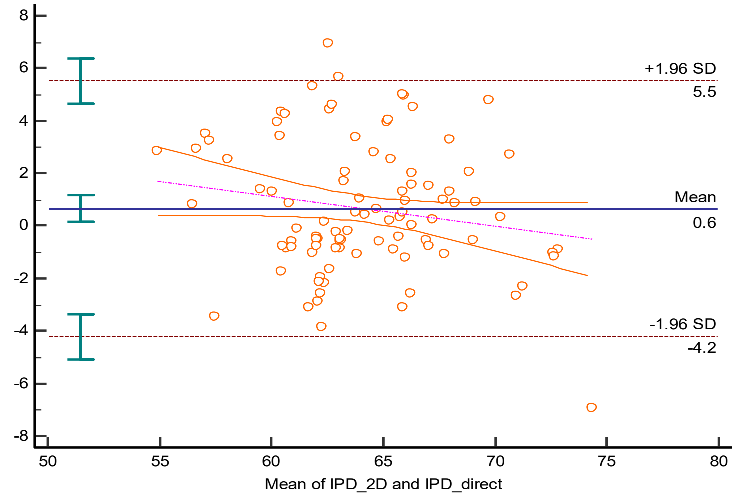

e-Figure 3e: Bland-Alman plot showed agreement between direct measurement and 2D photogrammetry for Interpupillary Distance and 95% limit of its agreement

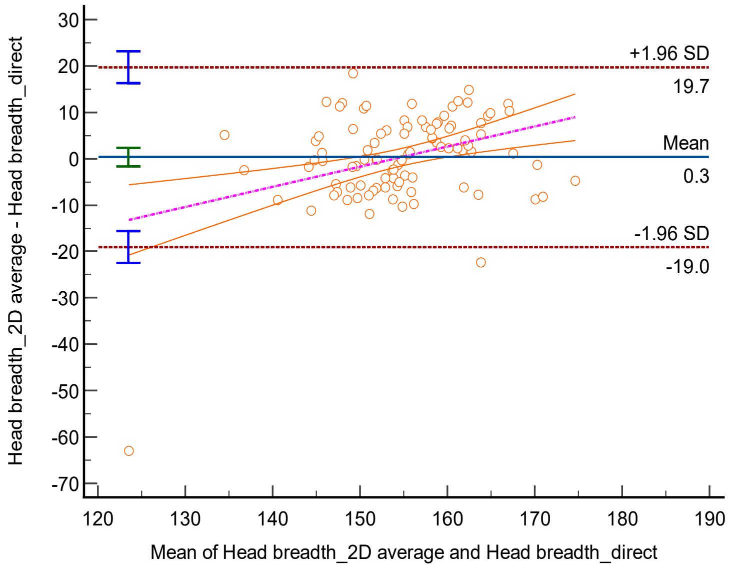

e-Figure 3f: Bland-Alman plot showed agreement between direct measurement and 2D photogrammetry for Head Breadth and 95% limit of its agreement

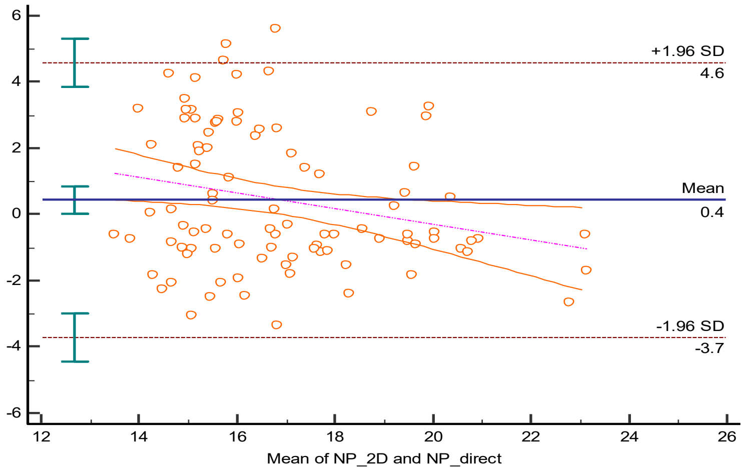

e-Figure 3g: Bland-Alman plot showed agreement between direct measurement and 2D photogrammetry for Nose Protrusion and 95% limit of its agreement

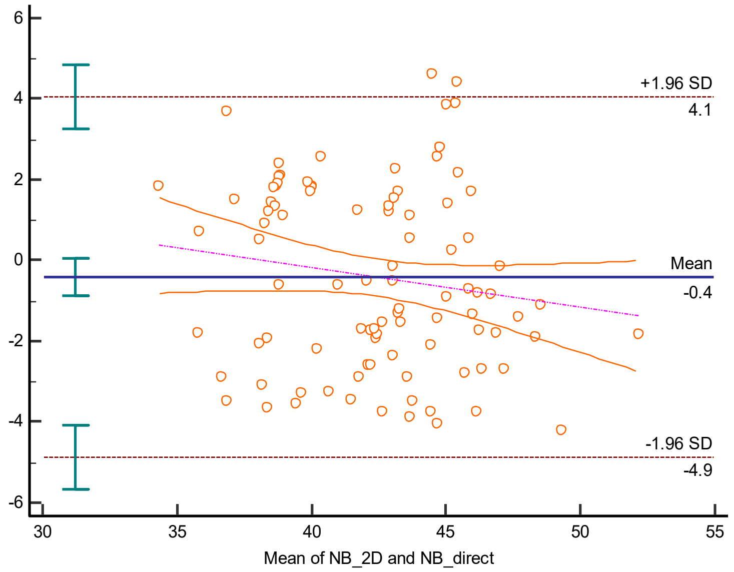

e-Figure 3h: Bland-Alman plot showed agreement between direct measurement and 2D photogrammetry for Nose Breadth and 95% limit of its agreement

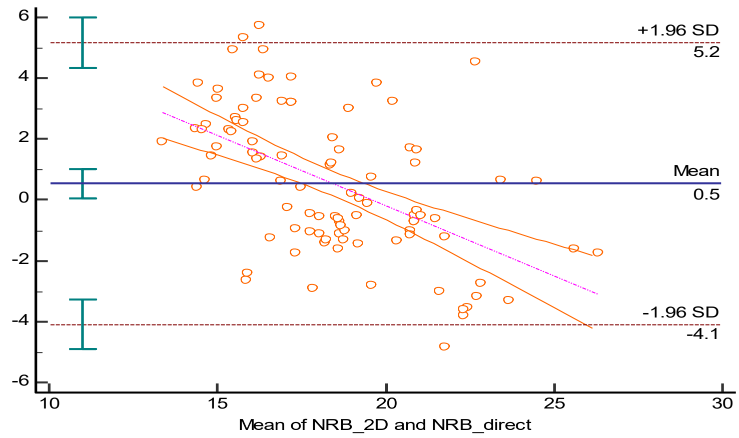

e-Figure 3i: Bland-Alman plot showed agreement between direct measurement and 2D photogrammetry for Nasal Root Breadth and 95% limit of its agreement

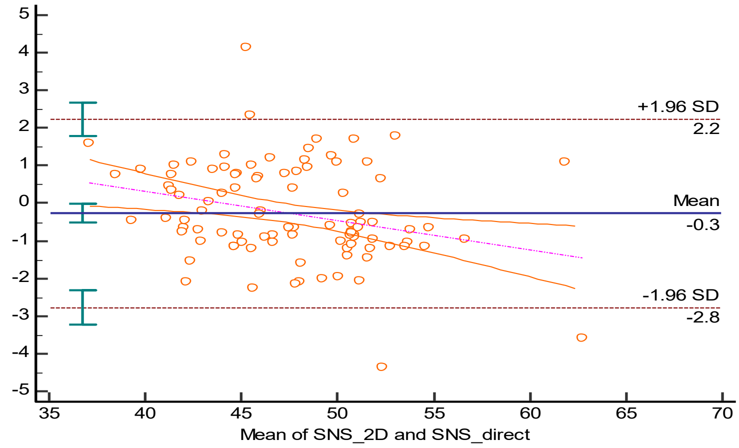

e-Figure 3j: Bland-Alman plot showed agreement between direct measurement and 2D photogrammetry for Subnasal-sellion Length and 95% limit of its agreement

e-Figure 3 Bland-Alman plot showed agreement between direct measurement and 2D photogrammetry, and 95% limit of agreement
